# Can’t Look Away: An Eye-Tracking Based Attentional Disengagement Training for Depression

**DOI:** 10.1007/s10608-016-9766-0

**Published:** 2016-03-16

**Authors:** Gina R. A. Ferrari, Martin Möbius, Amras van Opdorp, Eni S. Becker, Mike Rinck

**Affiliations:** 1 Behavioural Science Institute, Radboud University Nijmegen, PO Box 9104, 6500 HE Nijmegen, The Netherlands; 2 Pro Persona, Institute for Mental Health Care, Nijmegen, The Netherlands

**Keywords:** Attentional bias modification, Eye-tracking, Attentional disengagement, Depression, Stress

## Abstract

To address shortcomings of purely reaction-time based attentional bias modification (ABM) paradigms, we developed an ABM task that is controlled by eye-tracking. This task allows to assess and train both disengagement from negative pictures and maintained attention to positive pictures. As a proof-of-principle study with an unselected student sample, this positive training (PT; N = 44) was compared to a negative training (NT; N = 42), which reinforced the opposite attentional pattern. Importantly, training trials were completed only if participants performed the correct gaze patterns. Results showed that higher depression levels were associated with slower disengagement from negative stimuli at baseline. As expected, the PT induced longer fixations on positive pictures and faster disengagement from negative pictures. The NT showed no changes in attentional processes. The groups did not differ in mood reactivity and recovery from a stressor. Advantages of using eye-tracking in ABM and potential applications of the training are discussed.

## Introduction

Probably everybody recognizes the situation in which one simply cannot look away from a horrible movie scene or a car accident on the highway. Attending to such negative scenes does not make us happy, obviously, but we often cannot disengage our attention from it. In fact, from an evolutionary perspective, it makes sense to not completely ignore such potentially harmful situations or stimuli, in order to be able to keep the distance or to avoid them in the future (Rubenking and Lang 2014).

However, it would become maladaptive if we persistently attended to negative information at the expense of positive information; a tendency often found in individuals suffering from depression (Peckham et al. 2010). Beck (1976) postulated that this tendency can be explained by negative schemata which guide information processing in depressed individuals, leading them to selectively attend to negative, schema-congruent stimuli in their environment. According to Beck´s depression model (1976, 1987) as well as other prominent cognitive theories of depression (Teasdale 1988), such attentional processing biases play a causal role in the development and maintenance of the disorder.

Importantly, research suggests that the nature of this bias is different from the one reported in anxiety disorders (Gotlib and Joormann 2010). Unlike anxious individuals who show both an orienting bias towards threat (for reviews, see Cisler and Koster 2010; Mogg and Bradley 1998), as well as delayed disengagement from threat (Armstrong and Olatunji 2009; Fox et al. 2002; Schofield et al. 2012), the depressed do not seem to be more vigilant for negative stimuli than healthy individuals (Caseras et al. 2007; Sanchez et al. 2013). Instead, the attentional processing of negative stimuli in depression seems to be specifically characterized by the increased maintenance of eye-gaze on negative information (for a meta-analysis, see Armstrong and Olatunji 2012), supposedly reflecting a difficulty to disengage attention from it, once it has become the focus of attention (Gotlib and Joormann 2010). Although a number of eye-tracking studies provide evidence for the prolonged processing of negative information (see Caseras et al. 2007; Eizenman et al. 2003; Kellough et al. 2008; Sears et al. 2010), until recently it has remained speculative whether the maintained eye-gaze on negative information indeed is due to disengagement difficulties (Sanchez et al. 2013).

The most direct evidence for this assumption comes from research by Sanchez et al. (2013). In a novel engagement-disengagement assessment task employing eye-tracking, participants had to disengage their attention from negative pictures in order to attend to neutral pictures. Compared to healthy controls, depressed individuals took longer to disengage attention from negative stimuli when prompted to. Moreover, the depressed group lacked the positive attentional bias (i.e., longer sustained attention to positive than to neutral or negative stimuli) typically found in healthy samples (Armstrong and Olatunji 2012; Ellis et al. 2010; Kellough et al. 2008; Sears et al. 2010), indicating that processing of both negative and positive information is affected in depression.

Besides the disturbed disengagement from negative cues, depression has also been associated with a lack of inhibitory control over negative information (De Raedt and Koster 2010) and with the use of maladaptive emotion regulation strategies (e.g., rumination). This prevents individuals from implementing more effective strategies, such as redirecting attention away from negative stimuli and towards other, more benign cues, or reappraising emotion-eliciting situations in a less negative way (Gotlib and Joormann 2010; Joormann and D’Avanzato 2010). This in turn may lead to prolonged negative affect in response to stress. In line with the assumption that difficulties in disengagement are associated with impaired emotion regulation, Sanchez et al. (2013) showed that it was specifically impaired disengagement from negative stimuli which predicted impaired recovery from a stressful speech task.

Supporting the causal role of an attentional bias in depression and emotional vulnerability, a range of attention bias modification (ABM) studies have been conducted. They provide first evidence that training attention away from negative information and towards neutral or positive information decreases depressive mood and cortisol response to stress in dysphoric individuals (Tsumura et al. 2012) as well as depressive symptoms in dysphoric adults, adolescents, and remitted depressed individuals (Browning et al. 2012; Wells and Beevers 2010; Yang et al. 2014).

Most of these studies applied the task most commonly used to measure and modify attentional bias: the dot-probe task (MacLeod et al. 2002). In this task, individuals are exposed to series of consecutively presented pairs of negative and neutral or positive stimuli (images or words) on a computer screen. Participants are required to respond to a target stimulus (i.e., a probe), which always appears in the location of one of the two stimuli. Shorter reaction times to probes in the location of negatively valenced stimuli compared to neutral (or positive) stimuli indicate an attentional bias towards negative information. In the training version of the task, the probe occurs in about 85–100 % of the trials in the location of the neutral or positive stimulus, such that participants learn to attend towards the relatively positive information and away from negative information.

Despite early promising findings, an increasing number of studies failed to successfully modify selective attention with the dot-probe task and also failed to replicate its beneficial therapeutic effects (for reviews see, Cristea et al. 2015; Mogoase et al. 2014). A possible reason for the inconsistent findings might be the low reliability of the dot-probe task, which is related to the exclusive use of reaction time data when assessing attentional bias (Brown et al. 2014; Schmukle 2005; Staugaard 2009; Waechter and Stolz 2015). Another frequently provided explanation is that during the task, participants may completely ignore the emotionally valenced stimuli and only initiate their search for the probe once it is presented (Bradley et al. 2010; Notebaert et al. 2015), making it difficult to measure a possibly existing (disengagement) bias. Moreover, it has been suggested that for a change in bias to occur, it might be necessary for participants to detect the link between stimulus valence and probe location (Notebaert et al. 2015). Therefore, variations in the degree to which participants really attend to and hence process the stimuli might also explain the inconsistent training effects. Finally, it is important to note that the suitability of the dot-probe task has especially been doubted in the context of depression, as the task does not seem to allow for thorough conclusions about what is actually measured and targeted (Leyman et al. 2007). With longer stimulus durations, participants may shift their attention back and forth between the stimuli, leaving undetected which attentional component the task is tapping into: Heightened vigilance for negative stimuli or, more relevant for depression, impaired disengagement from negative stimuli.

These arguments suggest that conventional reaction-time based ABM paradigms such as the dot-probe task are not the most optimal procedures for measuring and modifying attentional bias, and particularly not so for depressed individuals (Mogoaşe et al. 2014). Researchers in this field have therefore repetitively stressed the need to further refine existing ABM tasks and to develop new theory-driven approaches for modifying attentional bias (Clarke et al. 2014; Mogoaşe et al. 2014). In a recent meta-analysis by Mogoaşe et al., the authors concluded that such future ABM procedures should not only aim to reliably modify selective attention, but that they should also have greater ecological validity and be more captivating for participants. Despite the growing acknowledgement of the need to develop new improved methodologies that go beyond the conventional dot-probe task, there are not enough studies on novel ABM paradigms yet (Clarke et al. 2014). A notable exception is the “person-identity-matching” (PIM) task recently proposed by Notebaert et al. (2015). This ABM paradigm was designed to modify the attentional bias that is characteristic for anxiety, and it may be a promising alternative to the conventional dot-probe task. However, it does not allow to target the attentional disengagement deficit found in depression. Moreover, change in bias was again assessed with the dot-probe task, and no reliable alternative for measuring training effects was presented by Notebaert et al. (2015).

Therefore, the aim of the current study was to address limitations of previous ABM paradigms and to develop and evaluate a novel, eye-tracking-based (ET) ABM paradigm to assess and target the disturbed attentional components operating in depression. In this proof-of-principle study, an unselected sample of students completed one of two training versions. In the positive training (PT), participants were trained to disengage attention from negative pictures and shift it towards positive pictures, and to maintain attention on positive pictures, despite the presence of negative pictures. In the negative training (NT), the opposite pattern was trained (i.e., disengagement from positive pictures and maintained attention on negative pictures). It should be emphasized that we aimed to develop a disengagement training because the findings of Sanchez et al. (2013) suggest that specifically the slowed disengagement from negative stimuli is associated with impaired mood regulation. In order to train disengagement from negative stimuli, it is, however, insufficient to only include disengagement trials, as this might induce a tendency to disengage attention from all pictures, regardless of their valence. Because of this, and because of the depression-specific lack of maintained attention to positive stimuli, trials were added where attention had to be kept on positive pictures. Notably, the training was completely controlled by participants’ eye-movements: Each trial could only be completed if the attentional pattern corresponding to the training condition was executed. This way, the pace of the task was perfectly tailored to each individual’s task performance. Changes in the two attentional components were assessed by eye-movement recordings during a modified free-viewing task.

To confirm the validity of our bias measure, we first investigated the relation of attentional bias with depressive symptoms. In line with previous research (Sanchez et al. 2013), we expected that higher baseline levels of depression would be related to slowed disengagement from negative stimuli. Given the unselected nature of our sample, the corresponding analyses should be considered exploratory, though. Regarding the training, we hypothesized that the PT would induce a positive attentional bias (i.e., relatively longer fixations on positive than on negative pictures) whereas the NT would induce a negative attentional bias. Moreover, we expected differential changes in the disengagement component of attention, namely that the PT group would become faster in disengaging attention from negative (towards positive) information, whereas the NT group would become slower in this process. Previous research on cognitive bias modification (CBM) procedures targeting approach-avoidance tendencies shows that training-induced changes in bias are only found in individuals being trained to avoid negative stimuli, but not in those being trained to exclusively approach positive stimuli (Ferrari et al. 2012). This suggests that CBM trainings with negative and positive stimuli might work specifically via increasing the avoidance of negative stimuli. Because of this and because our training was specifically developed to modify attentional disengagement, we did not have explicit expectations about changes in maintained attention.

Finally, to assess a causal link between attentional disengagement and mood recovery from stress, and to allow for validation of our training, participants completed a stress task at the end of the experiment. Based on previous research (Sanchez et al. 2013), we expected the PT group to show a higher recovery from the stressor than the NT group.

## Methods

### Participants

Seventy-eight female and 17 male students (mean age = 21.79 (SD = 5.15)) of Radboud University Nijmegen, the Netherlands, participated in return for course credit or a 15 Euro reward. Participants were assigned in a double-blind fashion to the PT (*n* = 48) or the NT (*n* = 47).

### Instruments and Materials

#### Baseline Questionnaires

To assess depression levels, the revised version of Beck’s Depression Inventory was used (BDI-II, Beck et al. 1996). The internal consistency of the BDI was good (*α* = .85). Moreover, to be able to control for possible differences between groups in trait anxiety and affect, two additional baseline questionnaires were administered. The trait subscale of the State-Trait Anxiety Inventory (STAI-T; Spielberger 1989) was administered to assess anxiety proneness, and the Positive and Negative Affect Scale (PANAS; Watson et al. 1988) was used to measure general levels of affect. The internal consistencies of these questionnaires in the current sample were excellent (STAI: *α* = .92) and good (PANAS subscales: PA: *α* = .86; NA: *α* = .89). All questionnaires were administered in the participants’ dominant language (German or Dutch).

#### Mood Ratings

To measure mood changes throughout the experiment, participants indicated on six items how they felt at the moment (0 = *not at all* to 10 = *very much*). The items *happy* and *sad* assessed general mood and were analyzed to investigate whether the training affected mood directly. A score for general mood was calculated by mirroring scores on the happy mood item and adding them to scores on the sad mood item, such that higher scores were indicative of more negative mood. Stress was measured by the items *content*, *relaxed*, *frustrated*, and *nervous,* which were analyzed to assess mood reactivity and recovery from the stressor. A stress score was calculated by mirroring scores on the items content and relaxed and adding those to the scores on the items frustrated and nervous. Consequently, higher scores reflected higher levels of stress.

### The ET-ABM Task

#### Stimuli

Recent research by Becker et al. (2016) showed that CBM trainings with a broad range of stimuli, which are not restricted to depression-relevant content (e.g., threatening stimuli), can effectively increase a positive processing bias and reduce emotional vulnerability in dysphoric students. Based on these findings, we decided to make use of a disorder-non-specific stimulus selection. Ninety positive and 90 negative pictures (14.3 cm × 10.7 cm) from different categories (e.g., people, animals, objects) were selected from the Nencki Affective Picture System (NAPS; Marchewka et al. 2014). Forty-five picture sets were created, always containing two positive and two negative pictures matched on content and absolute value of emotional valence (negative: *M* = 3.34, SD = 0.44; positive: *M* = 3.23, SD = 0.44; *t* (178) = 0.21, *p* = .834). Negative pictures were slightly more arousing than positive pictures, though (negative: *M* = 5.92, SD = 0.4; positive: *M* = 5.11, SD = 0.46; *t* (178) = 12.74, *p* < .001). The pictures were arranged in a 2 × 2 grid, separating the screen into four equally sized quadrants, with the picture location (upper/lower and left/right part of the grid) being counterbalanced across trials. The stimuli were displayed on a black 36.5 cm × 27.5 cm computer screen (IIyama Vision Master Pro 450), with 1 cm distance between the pictures. Participants were seated about 60 cm away from the screen’s center.

#### Task Design

The task consisted of pre-assessment, training and post-assessment. On each trial, a white fixation cross appeared in the middle of one of the four quadrants of the grid. After fixation of the cross for 500 ms, it disappeared and a set of 4 pictures appeared. By placing the fixation cross into one of the quadrants (instead of the screen center) and making sure that it was indeed fixated, we could reliably manipulate which of the 4 pictures was fixated first. The training contained two different types of trials: *negative* trials and *positive* trials. In the PT, participants had to disengage attention from negative pictures and shift it to positive pictures, and to maintain attention on positive pictures. On negative (PT: *disengagement*) trials, one of the two negative pictures replaced the fixation cross and participants had to look away from it and fixate one of the two positive pictures for 1000 ms. A fixation time of 1000 ms was chosen based on prior research, indicating that attentional bias in depression is only observed at longer stimulus durations (i.e., >1000 ms; De Raedt and Koster 2010). Upon a sufficiently long fixation of a positive picture, all pictures disappeared and a probe (i.e., an arrow pointing left or right, with the direction being counterbalanced across picture valence) replaced the previously fixated positive picture. Participants had to react to the arrow’s direction by pressing a computer key. The probe then disappeared and a new trial started. On positive (PT: *maintained attention*) trials, a positive picture replaced the fixation cross and the trial continued only if participants kept looking at this picture for 1000 ms, or if they fixated the other positive picture for 1000 ms. In the NT, the opposite pattern was trained: When a positive picture replaced the fixation cross (positive trial), participants had to look away from it and fixate a negative picture. When a negative picture replaced the fixation cross (negative trial), participants had to keep looking at this picture or fixate the other negative picture. Importantly, in both the PT and the NT, the participants’ gaze pattern controlled the appearance of the probe: As soon as a positive (PT) versus negative (NT) picture was fixated for 1000 ms, the probe replaced the fixated picture. However, participants were not told that their viewing patterns would influence the continuation of trials or the location of the arrows. The training contained 270 training trials distributed across 3 blocks, during which each of the 45 picture sets was presented 6 times, in a new random order for each participant.

**Fig. 1:**
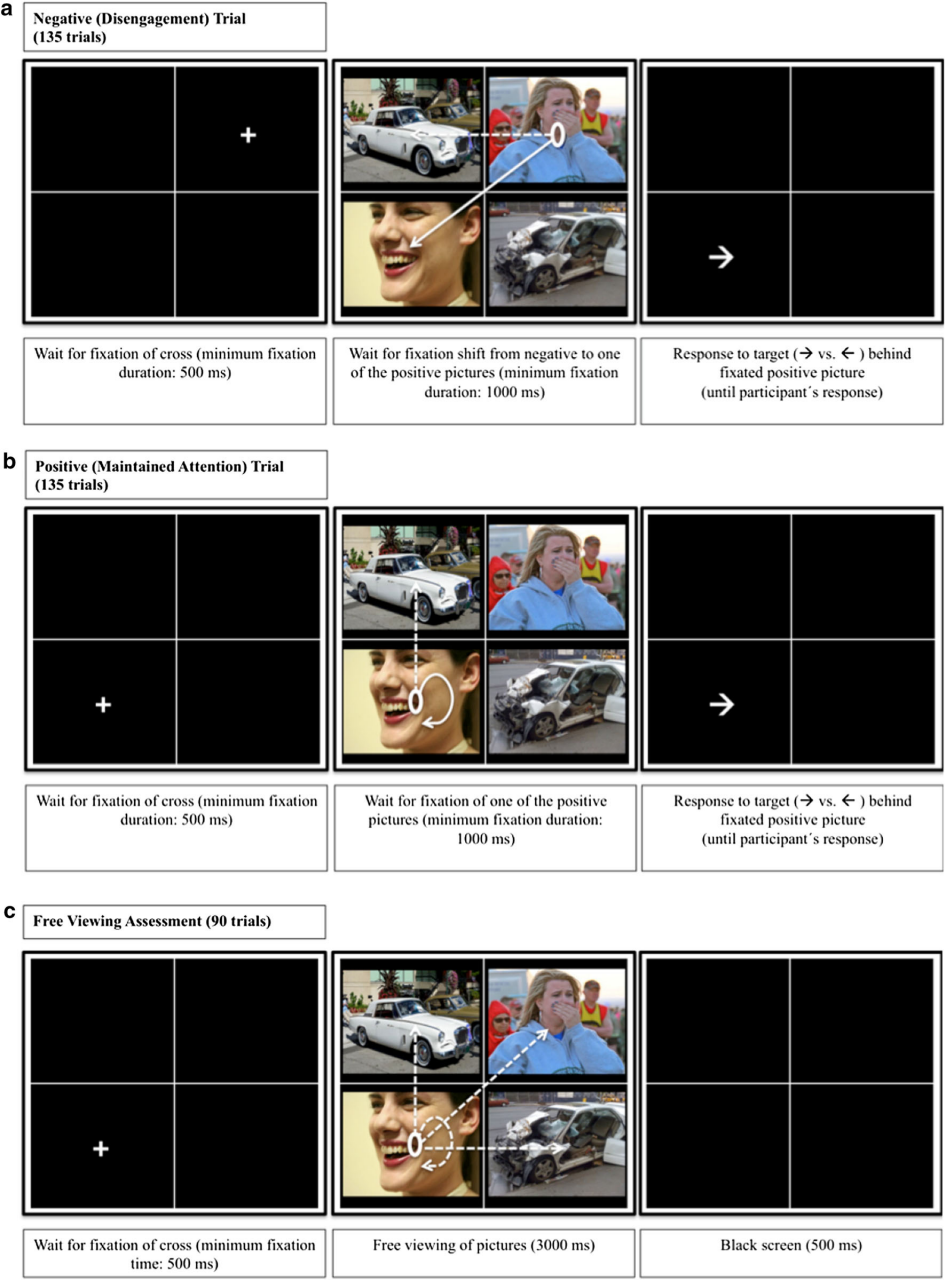
Schematic overview of the task design. On each trial of the Positive Training (PT), a fixation cross is presented. Upon fixation (500 ms), two negative and two positive pictures appear. **a** On negative (PT: disengagement) trials, participants have to disengage their attention from the fixated negative picture and fixate one of the two positive pictures. **b** On positive (PT: maintained attention) trials, attention has to be maintained at the fixated positive picture or at the other positive picture. **a**, **b** Upon fixation of a positive picture for 1000 ms, all pictures disappear and an arrow replaces the fixated picture. Participants respond to arrow direction by pressing a key. The arrow then disappears and a new trial starts. During the Negative Training (NT) not shown here, exactly the opposite attentional patterns are reinforced. **c** The free viewing task (assessment) is similar to the training, however, all trials last 3000 ms and no probe is presented. *Note*. This figure contains sample images, which have not been used in the current study. All images were obtained from Flickr and were published under a Creative Commons license. The formats of the images were slightly adapted for this figure. Credits: *top left*, Joe deSousa, CC0 1.0; *top right*, West Point—The U.S. Military Academy, CC BY 2.0; *bottom left*, Steven Depolo, CC BY 2.0; *bottom right*, bettyx1138, CC BY 2.0. For license terms see, CC0 1.0 (https://creativecommons.org/publicdomain/zero/1.0/); CC BY 2.0 (https://creativecommons.org/licenses/by/2.0/)

The pre- and post-assessment was introduced as a calibration procedure, and consisted of a free-viewing task similar to the training. However, independently of participants’ viewing patterns, all picture sets were presented for 3000 ms and no probe followed. During each assessment, the 45 picture sets were presented twice (90 trials), once as positive (PT: maintained attention) and once as negative (PT: disengagement) trials. During pre- and post-assessment, the location of the fixation cross was counterbalanced across valences and grid positions. The whole task took approximately 30 min. Figure 1 illustrates the task design.

#### Eye-Tracking Device

Monocular gaze data of the dominant eye were obtained at a frequency of 500 Hz, by means of the iView × Hi Speed system from SMI, a video based eye-tracking system.

#### Calculation of Attentional Indices

Fixation data recorded during the assessments were used to calculate the fixation time as an index of attentional processing. In accordance with Sanchez et al. (2013), only measurements where participants fixated a picture for at least 100 ms were considered. This index was used to calculate first, a “sustained attention bias” score, reflecting the proportion of total fixation time on positive compared to negative pictures, and second, the two attentional components relevant here: disengagement from negative pictures (short: negative disengagement) and maintained attention for positive pictures (short: positive maintained attention), separately for pre- and post-assessment.

For the sustained attention bias score, two sum scores were calculated for each trial, reflecting the total time participants fixated positive and negative pictures. Based on these scores, medians were calculated, representing the median time participants fixated positive and negative pictures.^1^ The bias score was then calculated as follows: (Median fixation time on positive pictures)/(median fixation time on positive pictures + median fixation time on negative pictures). Scores larger than 0.5 reflect a more positive sustained attention bias (relatively longer fixations on positive pictures), while scores smaller than 0.5 reflect a more negative sustained attention bias.

For the two attentional components, a median score was calculated for each trial type, representing the latency of the attentional shift from the first fixated picture to a picture of the opposite valence (until its fixation). On negative trials, shorter latencies of first shifts to positive pictures reflect faster negative disengagement. On positive trials, longer latencies of first shifts to negative pictures reflect longer positive maintained attention.

#### Stress Task

The task was adapted from Amir et al. (2008). Via the computer, participants were informed that they would get 1 min to prepare a 3-min-speech on the topic “nuclear power”, which would be video-recorded for later evaluation by two independent researchers. Participants were not allowed to take notes during preparation and a clock on the computer screen signaled the time left. After 1 min, a “beep” sound occurred. The experimenter entered the room, started the video-recording, asked participants to deliver their speech into a webcam, and left. After 3 min, the experimenter entered and stopped the video recording. Thereafter, participants rested for 5 min.

### Procedure

After providing informed consent, participants were randomly assigned to the PT or NT, and they completed the baseline questionnaires and mood measures (i.e., Likert scales; T0: before pre-assessment). Afterwards, they were seated in front of the eye-tracker. After determining participants dominant eye and running a brief calibration procedure, the assessment took place, followed by the training and another mood measure (T1: after training). Thereafter, the post-assessment followed. The calibration of the eye-tracker was repeated before each training block and before the post-assessment. After the post-assessment, participants completed the mood measures again (T2: before stress), and they took the stress task. Mood scales were again administered after providing the speech instructions (T3: anticipatory stress), as well as after speech delivery (T4: after stress) and the 5-min resting period (T5: recovery). To restore positive mood, a brief happy movie clip (Jungle book) was shown, followed by a final mood measure. Thereafter, participants filled in an awareness check, they were paid or received course credits, and they could leave behind their e-mail address for later debriefing. The experiment took about 90 min.

### Statistical Analysis Plan

To investigate group differences in attentional processes at baseline, an analysis of variance (ANOVA) was performed on the sustained attention bias scores, as well as a multivariate analysis of variance (MANOVA) on the two attentional components, negative disengagement and positive maintained attention. Moreover, Cronbach’s alpha and Spearman-Brown reliability coefficients were calculated separately for the three eye-tracking indices at baseline, to examine the reliability of our novel ET-ABM task.

To explore whether baseline depression levels would be related to late disengagement from negative information, exploratory correlations were computed between BDI scores and the attentional component variable, negative disengagement. Although we had no explicit expectation regarding the association of baseline depression levels with the second attentional component, positive maintained attention, we explored this correlation as well.

For the evaluation of training effects on attentional processes, first a 2 (group: PT, NT) × 2 (time: pre-training, post-training) repeated-measures (RM) ANOVA was conducted on the sustained attention bias scores. Second, for the effects on the two attentional components, a 2 (group: PT, NT) × 2 (time: pre-training, post-training) × 2 (trial type: negative trial, positive trial) RM ANOVA was performed. To additionally explore whether initial tendencies in attentional processes were related to the training effects, dependent on training group, two stepwise regression models were tested. In the first model, baseline sustained attention bias, group, and the interaction of the two factors were entered into the regression model to predict change scores of sustained attention bias (i.e., post-scores minus pre-scores). In the second model, disengagement from negative pictures at baseline, group, and the interaction of these factors were entered to predict changes in negative disengagement (i.e., post-scores minus pre-scores).

Finally, training effects on mood were investigated in two steps. To test whether the training directly affected general (i.e., happy and sad) mood, a 2 (group: PT, NT) × 2 (time: pre-training, post-training) RM ANOVA was conducted. Then, a similar analysis was conducted with scores on the stress scales (i.e., content, relaxed, frustrated and nervous) at the four time points during the speech task (before stress, anticipatory stress, after stress, recovery), to investigate whether the training affected mood reactivity and recovery from the stressor.

## Results

### Preliminary Analyses and Group Characteristics

Four participants were excluded because, due to technical problems with the eye-tracker, they completed <75 % training trials. Five additional participants were excluded due to extreme responses on the baseline questionnaires or outlying data on the eye-movement indices (i.e., data points more than 1.5 interquartile ranges below the first or above the third quartile). Due to skewness of the data, the BDI scores as well as two attentional variables (negative disengagement and positive maintained attention) were log-transformed. In all following analyses, Greenhouse-Geisser corrections were applied when the assumption of sphericity was violated.

The groups (PT: *N* = 44; NT: *N* = 42) did not differ significantly on the demographic variables or baseline trait characteristics (see Table 1).

**Table 1:** Group differences on demographic variables^a^ and Baseline Questionnaires

	PT (*N* = 44)	NT (*N* = 42)	
Age	21.45 (3.55)	22.44 (6.81)	*t*(83) = 0.84, *p* = .401
Gender			*ϰ* ^*2*^(1) = 0.85, *p* = .358
Male	7	10	
Female	37	32	
Nationality			*ϰ* ^2^(2) = 2.35, *p* = .309
Dutch	28	30	
German	16	10	
Education			*ϰ* ^2^(3) = 0.84, *p* = .84
Psychology	24	19	
Educational science	3	3	
Other	16	17	
No study	1	2	
Year of education	2.09 (1.39)	1.73 (1.47)	*t*(83) = 1.16, *p* = .25
BDI	5.93 (5.8)	5.29 (4.17)	*t*(84) = 0.59, *p* = .556
STAI-T	38.36 (9.96)	37.52 (10.07)	*t*(84) = 0.39, *p* = .698
NA	17.11 (6.67)	17.17 (6)	*t*(84) = 0.04, *p* = .969
PA	31.86 (6.33)	30.45 (5.76)	*t*(84) = 1.08, *p* = .284
Mood baseline	18.52 (7.22)	20.12 (7.07)	*t*(84) = 1.07, *p* = .289

*PT* positive training; *NT* negative training; *BDI* revised becks depression inventory (BDI-II); *STAI-T* Spielberger trait anxiety inventory; *PA* positive affect; *NA* negative affect

^a^Demographic information of two participants in the NT group was missing

### Attentional Processes at Baseline

#### Sustained Attention Bias

A one-way ANOVA revealed that the groups did not differ from each other on their sustained attention bias at baseline, *F*(1,84) = 2.24, *p* = .138. For means, see Table 1. A subsequent one-sample *t* test showed that, across groups, the pre-existing bias score (*M* = 0.5, SD = 0.05) did not differ significantly from 0.5 (*t*(85) = 0.56, *p* = .58), indicating that there was no sustained attention bias, neither for positive nor for negative pictures.

#### Negative Disengagement and Positive Maintained Attention

A multivariate analysis of variance (MANOVA) of the two log-transformed attentional variables revealed that the groups did not differ from each other in negative disengagement or positive maintained attention, *F*(2, 83) = 0.06, *p* = .946. An additional paired-samples *t* test, comparing the two attentional indices across groups, showed that participants took longer to disengage attention from negative pictures (*M* = 638, SD = 195) than from positive pictures (*M* = 575, SD = 159), *t*(85) = 4.32, *p* < .001. For means, see Table 2.

**Table 2:** Mean fixation times (with standard deviations) in milliseconds during the free viewing task, and the resulting attentional bias scores

	PT		NT	
	Pre-training	Post-training	Pre-training	Post-training
Fixation time on positive pictures	1416 (187)	1806 (444)	1377 (170)	1401 (334)
Fixation time on negative pictures	1353 (174)	936 (323)	1406 (170)	1421 (327)
Sustained attention bias score	0.51 (0.05)	0.65 (0.12)	0.49 (0.05)	0.5 (0.11)
Disengagement from negative pictures	622 (151)	542 (164)	655 (233)	706 (295)
Maintained attention for positive pictures	568 (146)	607 (216)	582 (172)	623 (262)

*PT* positive training; *NT* negative training; Sustained attention bias score: Proportion of total fixation time on positive pictures compared to negative pictures; Disengagement from negative pictures: Latency of the first shift from a negative picture until fixation of a positive picture; Maintained attention for positive pictures: Latency of the first shift from a positive picture until fixation of a negative picture

#### Reliability of the Attentional Process Measures

Cronbach’s alpha was calculated separately for all three eye-tracking indices at baseline. Trials with a large number of missing data (>10 %) were excluded (i.e., sustained attention bias: 2; negative disengagement: 8; positive maintained attention: 5). The resulting Cronbach’s alpha values for the three eye-tracking variables were good (sustained attention bias: *α* = .88; negative disengagement: *α* = .81; positive maintained attention: α = .79). Spearman-Brown reliability coefficients for the three indices were somewhat lower with .78 (sustained attention bias), .73 (negative disengagement), and .65 (positive maintained attention), but can still be considered acceptable.

### Relation Between Negative Disengagement and Depression

The correlational analysis revealed the expected significant correlation between BDI and negative disengagement latencies, *r*(86) = .23, *p* = .036, supporting that higher levels of depression were associated with slower disengagement from negative stimuli. By contrast, there was no evidence for a relation between depression levels and positive maintained attention (*r*(86) = .17, *p* = .126). Given the strong correlation between depression levels and anxiety levels in our sample, *r*(86) = .72, *p* < .001, we additionally explored the correlation between BDI and negative disengagement, while controlling for STAI scores. The correlation between BDI and negative disengagement became non-significant (*p* = .514). Likewise, the correlation between STAI scores and negative disengagement was non-significant when controlling for depression scores (*p* > .197).^2^

### Training Effects on Attentional Processes

#### Changes in Sustained Attention Bias

The 2 (group: PT, NT) × 2 (time: pre-training, post-training) repeated-measures ANOVA revealed significant main effects of time, *F*(1, 84) = 32.58, *p* < .001, *η*^*2*^ = .28, and of group, *F*(1, 84) = 34.46, *p* < .001, *η*^*2*^ = .29, which were subsumed under a significant time-by-group interaction, *F*(1, 84) = 31.24, *p* < .001, *η*^*2*^ = .27. In line with the training contingency, the PT group showed an increase in positive sustained attention bias, *t*(43) = 8.16; *p* < .001, whereas no change was found in the NT group, *t*(41) = 0.08; *p* = .935). An independent samples *t* test showed that the PT group had a more positive sustained attention bias after the training than the NT (*t*(84) = 6.24, *p* < .001). One-sample *t* tests revealed the presence of a positive bias in the PT group after the training, that is, relatively longer fixations on positive than on negative pictures, *t*(43) = 8.28, *p* < .001, while no bias was present in the NT group, *t*(41) = 0.23, *p* = .823. For means, see Table 2.

#### Changes in Negative Disengagement and Positive Maintained Attention

To assess training effects on the two attentional components separately, a 2 (group: PT, NT) × 2 (time: pre-training, post-training) × 2 (trial type: negative trial, positive trial) RM ANOVA was conducted. This analysis revealed significant main effects of trial type, *F*(1, 84) = 11.23, *p* = .001, *η*^*2*^ = .12, as well as significant interactions for group-by-trial type, *F*(1, 84) = 10.62, *p* = .002, *η*^*2*^ = .11, and time-by-trial type, *F*(1, 84) = 7.11, *p* = .009, *η*^*2*^ = .08. Importantly, the analysis also revealed a significant three-way interaction of group-by-time-by-valence, *F*(1, 84) = 10.83, *p* = .001, *η*^*2*^ = .11, indicating that the two groups showed differential changes in negative disengagement and positive maintained attention. No other main effects or two-way interactions were significant (all *p* > .17). Subsequent paired samples *t* tests revealed that the training effects were driven by a significant decrease in disengagement latencies from negative pictures in the PT group, *t*(43) = 4.39, *p* < .001. Thus, the PT group became faster to look away from negative pictures and towards positive pictures. No other comparisons were significant, showing that the training did not change maintained attention for positive pictures in the PT group, *t*(43) = 0.9, *p* = .372, nor did attentional components change in the NT group (negative disengagement: *t*(41) = 0.85, *p* = .401; positive maintained attention: *t*(41) = 0.46, *p* = .646). Independent samples *t* tests showed that after the training, the PT group was faster to disengage from negative pictures than the NT group (*t*(84) = 2.9, *p* = .005), while the groups did not differ in their maintained attention on positive pictures (*t*(84) = 0.6, *p* = .949). Whereas the sample was initially faster to disengage attention from positive than from negative pictures, after training, this was still the case only after NT (*t*(41) = 3.49, *p* = .001). The pattern in the PT group reversed into relatively faster disengagement from negative pictures (*t*(43) = 2.99, *p* = .005).

#### Prediction of Training Effects

The first regression model was conducted to predict training-induced changes in sustained attention bias. The model was significant, *F*(2,83) = 18.26, *p* < .001; *R*^*2*^ = .31, indicating that lower initial bias scores were related to a larger increase in bias in response to the training, *β* = −.188, *p* = .046. Moreover, the increase in positive sustained attention bias was higher after PT than after NT, *β* = .551, *p* < .001.

The second regression model was conducted to predict training-induced changes in disengagement from negative stimuli. It was significant as well, *F*(2,83) = 9.28, *p* < .001; *R*^*2*^ = .18. As in the first model, slower disengagement from negative pictures at baseline was related to a stronger decrease in disengagement latencies in response to the training, *β* = −.3, *p* = .003. This effect was moderated by training, indicating that participants with longer disengagement latencies from negative pictures at baseline in the PT group showed a stronger decrease in these disengagement latencies, compared to participants in the NT, *β* = −.306, *p* < .001.

### Training Effects on Mood

#### Direct Effects on General Mood

To investigate whether the training directly affected participants´ mood, data of those participants who completed the general mood scales after the training (PT: *n* = 31, NT: *n* = 36) were analyzed.^3^ The mood data were subjected to a 2 (group: PT, NT) × 2 (time: T0 pre-training, T1 post-training) RM ANOVA, which revealed a significant time effect, *F*(1, 65) = 21.71, *p* < .001, *η*^*2*^ = .25, which was modulated by a significant time-by-group interaction, *F*(1, 65) = 5.78, *p* = .019, *η*^*2*^ = .08. Subsequent independent samples *t* tests revealed that the groups did not differ in general mood before the training (T0; PT: *M* = 5.97; NT: *M* = 6.39; *t*(65) = 0.38, *p* = .706) but that the NT group showed a more negative mood than the PT group after the training (T1; PT: *M* = 6.68; NT: *M* = 8.61; *t*(65) = 2.6, *p* = .012). Means and standard deviations are presented in Table 3.

**Table 3:** Mean mood ratings (with standard deviations) for all assessment points

	T0: Before pre-assessment	T1: After training	T2: Before stress	T3: Anticipatory stress	T4: After stress	T5: Recovery
PT						
General mood	5.97 (2.82)	6.68 (2.79)	7.7 (3.2)	8.11 (3.04)	8.7 (2.43)	8.5 (1.95)
Stress	11.61 (4.65)	12.94 (5.05)	14.09 (6.31)	20.82 (7.12)	19.18 (7.93)	17.3 (6.51)
NT						
General mood	6.39 (2.75)	8.61 (3.24)	8.1 (2.64)	8.33 (3.33)	8.08 (2.54)	8.25 (2.18)
Stress	14 (5.17)	15.44 (5.32)	15.13 (5.11)	21.67 (6.86)	19.56 (7.11)	17.33 (6.42)

*PT* positive training; *NT* negative training; general mood (items: happy, sad); stress (items: content, relaxed, frustrated, and nervous)

#### Effects on Mood Reactivity and Recovery in Response to Stress

To investigate whether the training affected participants´ stress responses, data of those participants who completed the stress scales after the training were analyzed (PT: *n* = 44, NT: *n* = 39). The groups did not differ in stress levels directly before the stress task, *t*(81) = 0.74, *p* = .417. To test whether the training affected mood responses to stress, a 2 (group: PT, NT) × 4 (time: T2 before stress, T3 anticipatory stress, T4 after stress, T5 recovery) RM ANOVA was conducted on the stress scores. The main effect of time was significant, *F*(2.26, 182.96) = 34.81, *p* < .001, *η*^*2*^ = .3. Inspection of the means showed that the stress task had its intended effects: stress ratings increased upon announcement of the task (T2 before stress: *M* = 14.58, T3 anticipatory stress: *M* = 21.22), and decreased afterwards (T4 after stress: *M* = 19.36, T5 recovery: *M* = 17.31). However, the crucial group-by-time interaction was not significant, *F*(2.26, 182.96) = 0.22, *p* = .828, indicating that the groups did not differentially react to or recover from the stressor.^4^ All means and standard deviations are presented in Table 3.

### Exploratory Analyses of Awareness of Training Contingency

The two groups did not differ in their awareness of the training contingencies (aware of contingency PT: *n* = 9, NT: *n* = 13, *ϰ*^2^(86) = 1.24, *p* = .265). Additional exploratory analyses were conducted to investigate whether the training effects reported above depended on participants’ awareness of the training contingencies. For this purpose, the factor contingency awareness was added to all analyses, revealing that it was unrelated to the training effects. This was observed for the sustained attention bias score, *F*(1, 82) = 1.14, *p* = .288, and for the training effects on the two separate attentional components, *F*(1, 82) = 2.33, *p* = .131. Moreover, training effects on general mood directly after the training did not depend on contingency awareness either (general mood: *F*(1, 63) = 0.14, *p* = .708). The same was true for effects on mood reactivity and recovery in response to the stressor (stress: *F*(2.3, 181.57) = 0.21, *p* = .84).

## Discussion

This study investigated a novel ABM paradigm based on eye-tracking, designed to assess and target the attentional components that are disturbed in depression: disengagement from negative stimuli and maintained attention to positive stimuli. Eye-tracking was used to measure attentional changes and, more importantly, for training purposes: Only correct eye-movements were reinforced, such that participants could only complete the training trials if they showed the required attentional viewing patterns.

First of all, we replicated earlier research (e.g., Sanchez et al. 2013), showing that higher levels of depression were specifically associated with slower disengagement from negative information, confirming the validity of our measurement. Next and more importantly, our results showed that the training successfully modified selective attention, with the PT inducing a positive bias. No such change was found in the NT. Notably, these general training effects were driven specifically by faster disengagement from negative pictures in the PT group, indicating that our training directly taps into the attentional processes that are disturbed in depression. Although the PT functioned to induce relatively longer fixations of positive than negative pictures, the training did not modify the initial maintained attention for positive pictures. Again, no changes were found in the NT group, suggesting that the training did not induce dysfunctional attentional processes in our unselected sample.

The finding that the PT worked specifically by training disengagement from negative pictures is consistent with earlier research (Ferrari et al. 2012), supporting the relevance of targeting the processing of negative stimuli in modifying cognitive biases. A possible explanation for why the training did not increase initial maintained attention for positive pictures might be the perceptual salience of negative stimuli (Rozin and Royzman 2001). On positive trials, the tendency to first quickly scan the remaining pictures of the display might have overruled the tendency to sustain attention on the already fixated positive picture. Although this remains speculative, of course, future research using this ABM training should take the determining role of the negative stimuli into account and investigate whether training with exclusively negative disengagement trials is equally or even more effective than training with both types of trial. An alternative explanation for the absence of training effects on maintained attention might be related to our temporal criteria that defined a fixation during training. In order to continue with a training trial, participants in the PT had to fixate a positive picture for 1000 ms. In fact, these 1000 ms might not be sufficient for promoting “longer” maintained attention for positive stimuli. Instead, we might have trained short maintained attention to positive stimuli. Our main goal was to modify attentional disengagement with this training paradigm, which is also a reason for why we had no specific hypothesis about changes in maintained attention. In future research, the maintained attention component might be more optimally addressed by increasing the required fixation duration on positive pictures.

Regarding the above-mentioned findings, it is important to note that before training, our sample did not show the positive attentional bias which is supposed to be typical for healthy individuals. Instead, we observed equally long fixations of positive and negative pictures. More surprisingly, our sample even exhibited a tendency to disengage attention from negative pictures more slowly than from positive pictures (i.e., a disengagement bias), which contradicts a large number of studies providing evidence for a positive bias in unselected samples (e.g., Ellis et al. 2010; Kellough et al. 2008; Sears et al. 2010). A possible explanation for this unexpected finding might be related to the differences in arousal levels of positive and negative pictures used in the ET-ABM task. Positive stimuli are usually rated as less arousing than negative stimuli, independent of their emotional valence or pleasantness (see for instance, Lang et al. 2005). Although we matched the pictures on valence intensity, this was also the case with our selected set of pictures. Importantly, research has shown that attentional disengagement is slower from highly arousing stimuli than from stimuli low in arousal, independent of stimulus valence (Vogt et al. 2008). The difference in arousal levels thus might be the reason for why even our healthy sample showed longer initial fixations on negative than on positive pictures. At the same time, this pre-existing negative bias might explain why we were not able to further increase dysfunctional attentional patterns with the NT. The finding however, that the ET-ABM could not only induce a general positive bias, but even reverse the pre-existing disengagement bias in the PT group, has important implications for its potential clinical application. Together with the finding that initial disengagement from negative stimuli predicted greater changes in this attentional component, these results suggest that particularly depressed individuals might benefit from this new ET-ABM.

The successful modification of attentional processes further allowed us to investigate mood changes. Although negative mood increased in both groups throughout the training (possibly due to monotonicity of the procedure), this increase was stronger in the NT group. Earlier research, which failed to find immediate training effects on mood state, concluded that ABM procedures may only serve to affect the way in which people respond to subsequent situations requiring the processing of emotional information (e.g., MacLeod et al. 2002). Our results suggest that mood can be directly altered as a function of training contingencies with, in our case, a more negative mood resulting from reinforcing dysfunctional attentional patterns. This discrepancy might be related to the fact that the NT group needed to actually attend to, and hence process, the negative stimuli in order to complete our task. By contrast, in the dot-probe task, it might be enough to peripherally process the picture’s valence. Hence, differences between the groups in the depth of processing of the negative stimuli might explain why we found differential changes in mood.

Despite the direct effects on general mood, the training did not differentially affect mood changes in response to the stressor. The PT group did not show more attenuated stress levels or recovered more quickly from the stressor than the NT group, as suggested by Sanchez et al. (2013) or by Tsumura et al. (2012). It is important to note, though, that our sample did not include clinically or sub-clinically depressed individuals, therefore the sample should be less affected by stressful situations, and hence less susceptible to a CBM-induced stress reduction. While some studies did find stress-attenuating effects in healthy samples (e.g., MacLeod et al. 2002), other research suggests that such favorable effects are restricted to emotionally vulnerable individuals (Becker et al. 2016). Although our study does not support the causal role of negative attentional bias, and specifically slowed disengagement from negative stimuli in mood reactivity and recovery from stress, we suggest that future research should apply this training to a sub-clinically depressed sample, before drawing firm conclusions about its therapeutic value.

In a recent paper reflecting on the increasing number of reported ABM failures, Clarke et al. (2014) stated that it is highly unlikely that the first ABM task ever developed (i.e., the dot-probe task) will turn out to be the most successful one in modifying selective attention. It appears even more unlikely that this task, which has primarily been developed to affect anxiety vulnerability (MacLeod et al. 2002), will be equally effective for depression, considering the different nature of the bias in this disorder (Gotlib and Joormann 2010). The few studies, which sought to modify attentional bias in depression have mainly tailored the dot-probe task to depressed samples by using longer stimulus presentation times. However, the different attentional components of maintained attention and disengagement of attention cannot be targeted and assessed unambiguously with this procedure (Mogoaşe et al. 2014).

We assume that the strong training effects on attention found in our study are related to methodological advantages of the ET-ABM paradigm over conventional reaction-time based ABM tasks. The ET-ABM combines both trials starting with the fixation of a negative picture and trials starting with the fixation of a positive picture with the continuous measurement of eye movements. This allows for a more reliable measurement of attention and, more importantly, for a separate assessment of disengagement from negative stimuli and maintained attention on positive stimuli. Because the eye-tracker allows for targeted reinforcement of these specific gaze patterns, we can be sure that the use of alternative, undesired search strategies is discouraged and that the pace of the task is tailored to participants’ individual learning speed. The latter may be of particular importance when the training is applied in cognitively impaired, depressed samples. Furthermore, our paradigm addresses the demand for novel ABM tasks that have greater ecological validity and are more engaging (Mogoaşe et al. 2014). Contrary to the dot-probe task, the ET-ABM contains more than two visual stimuli per trial, increasing the requirement of redirecting attention away from negative stimuli and towards positive stimuli. Also, we made use of an unspecific and diverse selection of stimuli, representative of stimuli encountered in everyday life, rather than only pictures of faces, for instance. Finally, controlling the task by eye-movements might increase the game-like character of the procedure and have beneficial effects on its acceptability, which is crucial when we aim to provide the training to individuals with low motivation. Future research should investigate whether this ET-ABM task is indeed more acceptable than conventional ABM paradigms, and whether it results in fewer drop-outs when providing more training sessions.

Despite these promising findings, several limitations have to be noted. As a proof-of-principle study, we conducted the experiment in an unselected student sample. Although our sample was characterized by a disengagement bias, the results cannot be generalized to a depressed population. In fact, average baseline depression levels were very low (*M* = 5.62, SD = 5.05), ranging from 0 to 28, with only 3 participants scoring above 13 (mild to moderate depressive characteristics). Considering that BDI scores lower than 14 reflect minimal levels of depression (Beck et al. 1996), this suggests that our sample represented a rather healthy control group instead of an unselected group. This limited variance in depression levels might have obscured meaningful relations between depression scores and measures of attentional processes. It also questions the interpretability of our findings regarding the association of heightened depression levels with slower disengagement from negative stimuli at baseline. Analyses involving BDI scores must therefore be interpreted with caution, as findings cannot easily be generalized to higher or even psychopathological levels of depression. The interpretability of the above-mentioned association is complicated even more by the high correlation of depression and anxiety levels in our sample, and by the fact that the association disappeared when anxiety was taken into account. Although this suggests that heightened levels of psychopathological traits are related to difficulties in disengaging attention from negative stimuli, we cannot identify the unique role of depression in this relationship.

Second, the lack of a proper sham-training control group limits the interpretation of our findings. Future research needs to compare the PT to a placebo training condition in order to test its beneficial effects on attentional processes. Third, the use of an identical picture set in both training and assessment does not allow for any conclusions regarding the transfer of training effects to other stimuli. It is also possible that we in fact did not change the attentional processing of negative and positive stimuli in general, but that the observed effects are restricted to the specific stimuli we used during training. Fourth, to further validate our training and to test its potential superiority over existing ABM tasks, such as the dot-probe task, research should directly compare the two paradigms. Moreover, employing an eye-tracker in the ET-ABM is obviously more expensive and more difficult than simply using a standard PC in conventional ABM tasks. Therefore, the cost-effectiveness of the ET-ABM needs to be determined and compared to simpler ABM tasks. Finally, as we assessed attentional processes only directly after training, it remains unclear how long the effects of a single training session will last. Especially for clinical improvement, an enduring change in attentional processes is crucial. We therefore recommend that future research should include multiple training sessions and long-term follow-up measures, to provide insight into the temporal stability of the modified attentional processes.

To conclude, this is one of the first studies that developed and tested a novel ABM task, targeted at the attentional processes which are biased in depression. A single session of this new ET-ABM could induce a sustained attention bias for positive information and increase disengagement from negative stimuli. Although we could not find evidence for effects of the training on stress responses, the association of slowed disengagement with higher depression levels at baseline suggests that repeated training sessions with this task might have therapeutic relevance. Considering the rather healthy status of our sample and the strong correlation with anxiety levels, which might also explain the above-mentioned relationship, this interpretation certainly has to be treated with caution. Future research is needed to confirm this preliminary finding, by replicating this study in a sample with elevated or subclinical levels of depression. With more research into novel ABM paradigms, we hope to get closer to finding the most effective way of producing enduring changes in attentional processes underlying depression.

## Footnotes

1 As the raw gaze latencies used to calculate the three attentional indices were not normally distributed, median scores were calculated rather than mean scores, in order to reduce the impact of skewness and extreme values in the raw data.

2 Given the unselected nature of our sample, we additionally explored the correlations between the two log-transformed attentional component variables and the other trait questionnaires (STAI and PANAS). While the correlations with PANAS were not significant (*p* > .133), anxiety ratings were positively related to maintained attention to positive stimuli, *r*(86) = .22, *p* = .046, as well as to disengagement from negative stimuli, *r*(86) = .25, *p* = .021, indicating that the more anxious participants were, the longer they took to disengage attention from the picture they had fixated first, irrespective of the picture's valence.

3 Due to technical and experimenter errors, data from only 67 participants were available for this analysis.

4 As Sanchez et al. (2013) found that difficulties in disengagement specifically predicted lower recovery from sad mood after stress, we conducted additional exploratory MANOVAs, with the change scores of all six mood items as dependent variables, for the different phases of the experiment. A MANOVA on the six mood change-scores from before (T0) to after the training (T1), revealed no significant effect of group, *F*(6, 60) = 1.63, *p* = .156. A MANOVA on mood changes in response to the speech task (mood reactivity: T2–T3) revealed no significant group effect either, *F*(6, 76) = 1.23, *p* = .3. Also, a MANOVA on change scores of all mood items, from announcement of the speech task (T3) to after the recovery phase (T5; mood recovery) was not significant, *F*(6, 76) = 0.31, *p* = .93.
